# A Novel Routing Protocol Based on Elliptical Shaped Movement of Autonomous Underwater Vehicles in Data Gathering Process for Underwater Wireless Sensor Network

**DOI:** 10.3390/s22145269

**Published:** 2022-07-14

**Authors:** Ghulam Ali, Tariq Ali, Insha Ul Hassan, Ahmad Shaf, Muhammad Irfan, Grzegorz Nowakowski, Kazimierz Kielkowicz, Adam Glowacz, Samar M. Alqhtani

**Affiliations:** 1 Department of Computer Science, COMSATS University Islamabad, Sahiwal Campus, Sahiwal 57000, Pakistan; ghulamali.cs@gmail.com (G.A.); inshahassan45@gmail.com (I.U.H.); ahmadshaf@cuisahiwal.edu.pk (A.S.); 2 Electrical Engineering Department, College of Engineering, Najran University, Najran 61441, Saudi Arabia; miditta@nu.edu.sa; 3 Faculty of Electrical and Computer Engineering, Cracow University of Technology, Warszawska 24 Str., 31-155 Cracow, Poland; gnowakowski@pk.edu.pl (G.N.); kazimierz.kielkowicz@pk.edu.pl (K.K.); 4 Department of Automatic Control and Robotics, Faculty of Electrical Engineering, Automatics, Computer Science and Biomedical Engineering, AGH University of Science and Technology, al. A. Mickiewicza 30, 30-059 Kraków, Poland; 5 Department of Information Systems, College of Computer Science and Information Systems, Najran University, Najran 61441, Saudi Arabia; smalqhtani@nu.edu.sa

**Keywords:** ESEDG, elliptical trajectory, gateway nodes, mobile nodes, energy consumption, delay efficient

## Abstract

High end-to-end delay is a significant challenge in the data collection process in the underwater environment. Autonomous Underwater Vehicles (AUVs) are a considerably reliable source of data collection if they have significant trajectory movement. Therefore, in this paper, a new routing algorithm known as Elliptical Shaped Efficient Data Gathering (ESEDG) is introduced for the AUV movement. ESEDG is divided into two phases: first, an elliptical trajectory has been designed for the horizontal movement of the AUV. In the second phase, the AUV gathers data from Gateway Nodes (GNs) which are associated with Member Nodes (MNs). For their association, an end-to-end delay model is also presented in ESEDG. The hierarchy of data collection is as follows: MNs send data to GNs, the AUV receives data from GNs, and forwards it to the sink node. Furthermore, the ESEDG was evaluated on the network simulator NS-3 version 3.35, and the results were compared to existing data collection routing protocols DSG–DGA, AEEDCO, AEEDCO-A, ALP, SEDG, and AEDG. In terms of network throughput, end-to-end delay, lifetime, path loss, and energy consumption, the results showed that ESEDG outperformed the baseline routing protocols.

## 1. Introduction

Water covers around 70.1% of the Earth’s surface. Rapid technological advancements have resulted in better methods for observing underwater conditions. Oceanographic data collection, assisted navigation, ocean sampling, catastrophe avoidance, pipeline surveillance for oil and gas, and mine detection can benefit from underwater wireless sensor networks (UWSNs) [1,2,3]. Due to the substantial attenuation and easy absorption of radio frequency (RF) signals in the water, the signals do not propagate well in the UWSNs. As a result, acoustic communication remains efficient only because of higher data rates [1]. However, the acoustic signals pose several design issues, such as undesirable channel interference that might occur over the network due to the data retransmission procedure, which might result in a large amount of overhead [4]. Bandwidth issues, propagation delays, high data error rates, long end-to-end delays, ineffective data collection, short network lifespan, continuous topology changes, and higher power consumption are all issues in UWSNs [5,6]. In an acoustic network, communication distance is minimal [7,8]. In past years, many routing techniques have been proposed to collect data using multihop routing [9,10,11,12,13]. However, multihop has many disadvantages such as more energy utilization by sensors, the long delay between sender node to sink node, and high PDR [14].

UWSN architecture consists of mobile sensor nodes that are deployed randomly or in grid topology. These sensor nodes are further divided into two groups: data gathering and sink nodes. Data gathering nodes are deployed underwater at different depths, include acoustic modems for communication, and are mobile. Sink nodes are placed at the water surface, include both radio and acoustic modems used for communication, and are static. In UWSNs, sensor nodes sense the data and send it toward the sink node by using multiple intermediate nodes.

Most UWSN applications require constant data gathering and transmission due to the unpredictable underwater environment. There is a mechanism in traditional data gathering for delivering sensed data in a multihop fashion to a sink node [3,15]. Autonomous underwater vehicles (AUVs) have been closely monitored to address this issue [9,14]. Before returning home, the AUV gathers data from all sensor nodes and sends it to the destination (sink) node by using the shortest path tree (SPT) [16]. These underwater vehicles use actuators, sensors, and onboard intelligence to collect data without human interaction [17,18]. The data collection procedure is the most critical aspect of UWSNs [19]. In recent work, data gathering with an AUV eliminates multihop transmission data, enhancing UWSN energy balancing [20]. To conserve sensor node transmission power, the AUV collects data from only a few nodes, known as gateway nodes (GNs) or route nodes, rather than visiting each node or cluster. The selection of GNs depends on the hello packets of received signal strength indicator (RSSI), node zones (closer nodes of AUV), and residual energy [21]. Member nodes (MNs) are associated with GNs by flooding data packets with the SPT method. When the GNs have consumed enough power, a new node with more residual energy from its neighbors is chosen as the gateway node.

The selection of the AUV path is crucial because by using that path, an AUV can collect maximum data from nodes and lower energy consumption. If the AUV does not travel consistently, considerable packet loss and fluctuating energy usage may occur [22]. Recent research indicates that an elliptical-shaped trajectory is the best choice for effective data collection; maximum time is required to associate those MNs with GNs, resulting in a sizeable end-to-end delay.

To overcome end-to-end delay with maximum data gathering problems, we have proposed the Elliptical Shaped Efficient Data Gathering (ESEDG) routing protocol. In ESEDG, an elliptical-shaped trajectory has been designed for AUV movement by considering the network area size. For data collection, all nodes have been categorized as member nodes (MN) and gateway nodes (GN). An MN makes a path with other MNs towards a GN by adopting a location-aware shortest path tree algorithm. ESEDG creates the maximum number of GNs and reduces the time required in the vertical association of the MN with the GN. Afterward, the AUV moves in an elliptical trajectory and collects data from the GN.

The rest of the paper is formatted as follows: Section 2 contains related work; the problem is defined in Section 3. Section 4 describes the system model, trajectory design, and delay models. The comparison of the proposed ESEDG algorithm and results are discussed in Section 5. In Section 6, the conclusion and future work are discussed.

## 2. Related Work

In [20], a routing technique called AEERP (AUV aided energy-efficient data-gathering routing protocol) in which an AUV collects data via gateway nodes based on RSSI values of hello packets was proposed. AEERP maximizes data collecting while consuming less energy and delivering more data. In AEERP, the AUV does not have dynamic path adjustment of the elliptical trajectory when the network size increases or decreases.

In [21], an AEDG routing scheme helps expand network lifetime and decrease data packet loss due to low energy consumption by using an AUV, which collects data from GNs and sends it to sink nodes. The AUV uses the SPT method to associate the least amount of MNs with the GNs based on residual energy, which helps to reduce data packet loss. The longer end-to-end delay still exists in AEDG.

In [9], the data gathering protocol (DGS) in which an AUV collects data from the nodes is categorized as essential and usual data as it passes through the network. The nodes not selected as GNs send average data to GNs closer to the AUV. The protocol improves packet delivery efficiency. However, nodes relative to the AUV consume energy more quickly than nodes further away, resulting in an uneven energy consumption network.

In [13], AEEDCO and approximate (AEEDCO-A) have been proposed to collect the maximum amount of data and increase network throughput time by consuming less energy and data delivery time to improve the network efficiency in UWSNs. Data collection from each cluster head makes the clusters less energy efficient and includes delay-tolerant routing protocols.

In [23], based on data collection, the AUV location predictor (ALP) system was created. In this approach, the AUV collects data by moving along a predefined trajectory, and the AUV directly receives data from closer nodes, while the rest of the nodes forward data to GNs. They are more relative to the trajectory. The nodes close to the trajectory become “hot regions”, meaning they use less energy to convey data to the AUV. In the ALP, there is no end-to-end delay model introduced for nodes to communicate with the AUV.

In [24], the authors introduce the Scalable and Efficient Data Gathering (SEDG) routing protocol, which optimizes the collaboration of MNs with GNs to increase data delivery packets while consuming less energy. Furthermore, it reduces the drop data packet ratio interval time and increases network throughput time. SEDG does not address the delay model. SEDG makes paths based on the shortest path tree algorithm without considering the location and status (alive or dead) of the node.

In [25], the Atomic Shaped Efficient Delay and Data Gathering (ASEDG) model, which achieved network performance indicators such as network delay, balanced energy consumption, and end-to-end delay, is examined. In [26], CARP uses connection quality information to forward packets. It uses basic topological information to prevent void and shadow zones. In [27], by using flood zone angel, the data is sent to sink nodes in UWSNs. Sensors forward packets based on flooding angle and energy condition.

In [28], an enhanced hydro cast with an AUV for data collection has been proposed. The pressure levels of sensor nodes are used to route traffic to the sink in a greedy multihop method. In [29], the network considers two key factors: data packet size and transmission power utilized by sensors. By considering these factors jointly, a system has been proposed that can increase the network life using integer linear programming. In [30], a technique for data collection process in which the AUV uses Dubin curves to collect data from various 3D targets is proposed. First, it transforms 3D targets into 2D ones and builds a 2D route. Second, it changes the 2D path into a Dubin curve using ERT (Euler rotation transformation).

In [31], two data-gathering schemes to reduce imbalanced energy usage and network delay time are proposed. They employ an AUV and multihop transmission. Furthermore, it also addresses how to convey more important data to sink nodes and how to extend the network’s life.

The AUV path is essential for complete data collection and to balance energy utilization by sensors in UWSNs. There is no standard mechanism for selecting the best path for AUV rotation to collect data and consume less energy. To fill this gap, an AUV-based pattern for selecting the best path and less energy consumption protocol has been proposed [10].

In [9], the authors propose an AUV-aided data gathering routing protocol (AEDG), which divides nodes into two categories: GNs (near the AUV) and member nodes (not near the AUV) (MNs). The GNs receive data from the MNs depending on RSSI values, and the GNs send collected data to a moving AUV. This routing technique increases packet delivery rates while reducing node energy consumption. The “hot region” problem is caused by the AUV’s fixed journey path, which means that nodes along the path consume energy more quickly.

## 3. Problem Definition

Most routing protocols for UWSN data transmission propose linking to sink (destination) nodes by connecting multiple transitional sensor nodes. In existing protocols, there is a big chance of network delay, unbalanced energy consumption, node failure, and inefficient data gathering. An AUV that rotates in a predefined trajectory was introduced to overcome these problems. The best choice for the path of the AUV to follow when data gathering is elliptical because the proposed rotation covers the maximum network area and produces the maximum number of GNs that can help maintain the energy balancing and network life. This trajectory, however, has some flaws.

1. One noteworthy challenge is to create a new trajectory for the following reasons:
  - The sensor’s nodes consume high levels of energy when setting up connected dominant set (CDS) nodes at the initial stage.
  - Sorting the CDS to build an elliptical design has a high cost of computing in terms of energy and time.
2. Fixed-size elliptical shape used for various network area sizes.
3. Backtracking and network delay exist because the SPT is applied only to a specified network area.

## 4. System Model

The network design, node deployment pattern, AUV movement design, functioning principles, and restrictions are all covered in detail. The classifications of nodes are based on a number of attributes.

1. The Member nodes (MN).
2. The Gateways nodes (GN).
3. An autonomous underwater vehicle.

Objective: The main objective of this research is to enhance the network lifetime and network throughput (minimize data loss). The network settings are as follows:

1. The GNs and MNs are randomly deployed in the network area and have four types of nodes; MNs, GNs, AUVs, and sink nodes. The AUV moves in a predefined trajectory, and the sink nodes reside on the water surface.
2. MNs collect data from their neighbor nodes and submit them to the GNs in the specified period. The GNs hand over the data to the AUV as it moves in a predefined trajectory. Finally, the AUV transmits the data packets to sink nodes.
3. Nodes update other nodes with their residual information via a packet header when sending information to each other.

There are some limitations of the proposed system model:

1. It does not perform well where the network area is irregular.
2. The AUV cannot move in a backward direction.
3. Without path association, member nodes cannot send data to gateway nodes.
4. It does not support clustering in the network.
5. It does not support the routing procedure of more than 350 sensor nodes.

### 4.1. Trajectory Design

We introduce a technique named the Elliptical Shaped Efficient Data Gathering (ESEDG) for maximum data gathering and to balance energy utilization and reduce network delay. With the aid of GNs, the ESEDG can gather a large amount of data and decrease the network’s end-to-end latency.

### 4.2. Existing Trajectory Design

**Figure 1:**
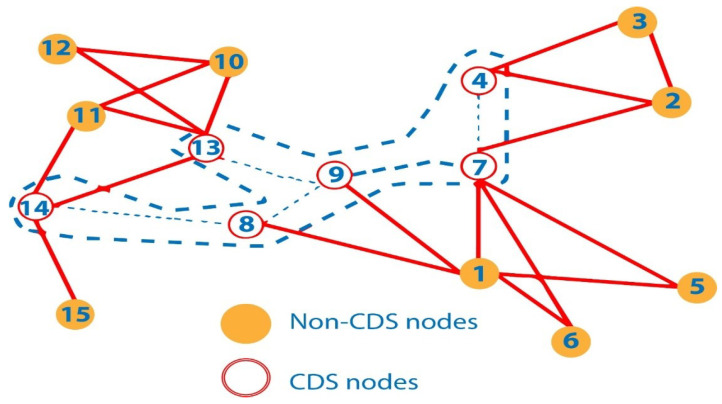
Sensor nodes identity represent in digits for AUV trajectory with HC.

With the help of connected dominant set (CDS) nodes, the existing elliptical-shaped trajectory of the AUV route was developed. The CDS is a set of interconnected nodes that gives more than one path to the sink node of a network. The minimum spanning tree (MST) of CDS nodes was calculated after the selection of CDS nodes, and the Hamilton circuit (HT) was established after the construction of MST, as shown in Figure 1. The CDS nodes were designed in a random shape and did not allow us the flexibility to build a complete trajectory. However, HC turned it into the correct circular or oval trajectory. This creates an elliptical trajectory that is not constrained by network size. Previous work has not addressed elliptical size ratio fluctuations during network expansion or shrinkage.

### 4.3. Proposed Trajectory Design

We proposed a horizontal elliptical AUV trajectory for UWSNs. In ESEDG, a node is deployed at the center of the network according to the network size. With the help of the central node, we can design different horizontal shapes by considering two variables, a and b. The X-axis covers the central axis, and the Y-axis of the elliptical trajectory covers the minor axis.

#### Defining Major and Minor Axes

**Figure 2:**
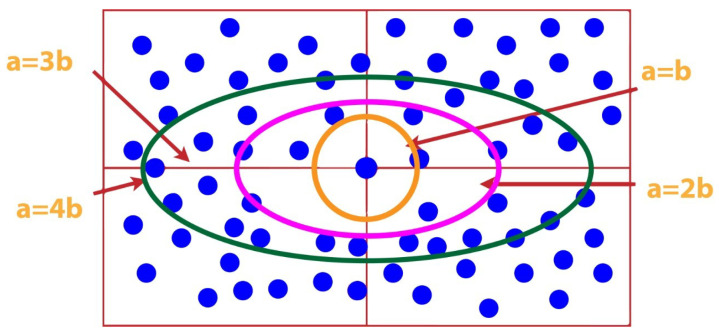
Multiple ellipse.

Figure 2 shows different elliptical designs by using the value of b static to define the major and minor axes.Blue dots represents sensor nodes in netwrok area.

The above procedure helps us design the horizontal elliptical shape or trajectory. Multiple trajectories can be created by using the center node of the network.

### 4.4. Delay Models

As we know, any network’s delay is an important characteristic to keep in mind to reduce the delay. Two delay models are discussed in this research.

1. MNs to MNs Delay.
2. MNs to GNs Delay.

### 4.5. MNs to MNs Delays

In networks, the delay means how long it takes for data to travel from the source node to the destination node. If the next forwarder is far from the source node, it may create forwarding delays. Due to this delay, there is a high possibility of data packet loss and more energy utilization by the nodes. To eliminate this issue, an algorithm was proposed to reduce the node-to-node delay for selecting the next forwarded ESEDG. All nodes are position aware, and one node deployed at the network’s center position plays a major role in choosing the next forwarder. A source node decides the next forwarder node’s direction based on the center node’s location.

**Figure 3:**
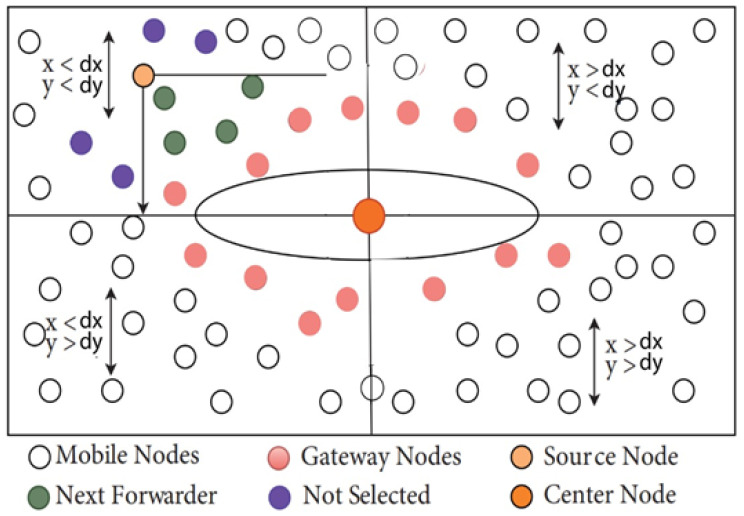
Selecion of next forwaded node.

In Algorithm 1, num represent all-sensor nodes of the network, and dx and dy represent the node center position. The algorithm is divided into four phases. A horizontal ellipse draws at the quadrant with X and Y-axes when a node selects a source node. The node status (“alive” or “dead”) is included in the algorithm and will help us find the next forwarded node. If the node’s status is “alive”, then it will save the numbers of that node, and the same node will be selected as the next forwarded node. The procedure will continue until the all-next forwarder nodes reach the GNs. This can help us increase the network life and lower energy use by the sensors. The dead nodes cannot be considered as the next forward node. The source node has four possible paths to the node because the network region has four quadrants as shown in Figure 3. Initially, the source node compares its distance from the centrally deployed node. Second, it analyzes the central node position to establish its bounds. In the third phase, neighbors receive an acknowledgment that includes their location when sending the HP to a node. The selection of the following forwarder is based on the distance between and sender and receiver node. For the next forwarder, two conditions must be met in all regions: the first is that they must be in the vicinity of the source node on either side of the X and Y axes, and the second is that the source node’s X or Y axis must be a boundary. There must be at least one node in the first region for the next forwarder to be greater than the source node in the first region’s axes. In the second area, the X and Y axes of the following forwarder must be more minor and more significant, respectively, than those of the source node. In the third region and the fourth region, the next forwarder must have a larger (X) and smaller (Y) dimension than the source node’s (X) and (Y) dimensions, respectively.

- First region: x≤dxandy≥dy;
- Second region: x≤dxandy≥dy;
- Third region: x≤dxandy≥dy;
- Fourth region: x≤dxandy≥dy.

**Algorithm 1:** Selection of next forwarded Node
1:num = network nodes2:s = nodes status3:*arr = new int(n)4:dx = Central node X – axis5:dy = Central node Y – axis6:**for**z=1 to num **do**7:   **if** s==”Alive” **then**8:     arr[]=z9:   **else**10:     Discard node z11:   **end if**12:
**end for**
13:arraySize = sizeof(arr)14:intSize = sizeof(arr[0])15:length = arraySize / intSize16:**for**i=1 to length **do**17:   a=ith node X−axis18:   b=ith nodeY−axis19:   **if** a≤dx and b≤dy **then**20:     Next forwarder (a,b++) ,(a++,b),(a++,b++)21:   **else if** a≥dx and b≤dy **then**
22:     Next forwarder (a,b++) , (a−−,b),(a−−,b++)
23:   **else if** a≤dx and b≥dy **then**
24:     Next forwarder (a,b−−) , (a++,b),(a++,b−−)25:   **else if** a≥dx and b≥dy **then**
26:     Next forwarder (a,dy−−) , (a−−,dy),(a−−,b−−)27:   **end if**28:
**end for**



By implementing these conditions, each region’s node can choose the next forwarder.

### 4.6. MNs to GNs

**Figure 4:**
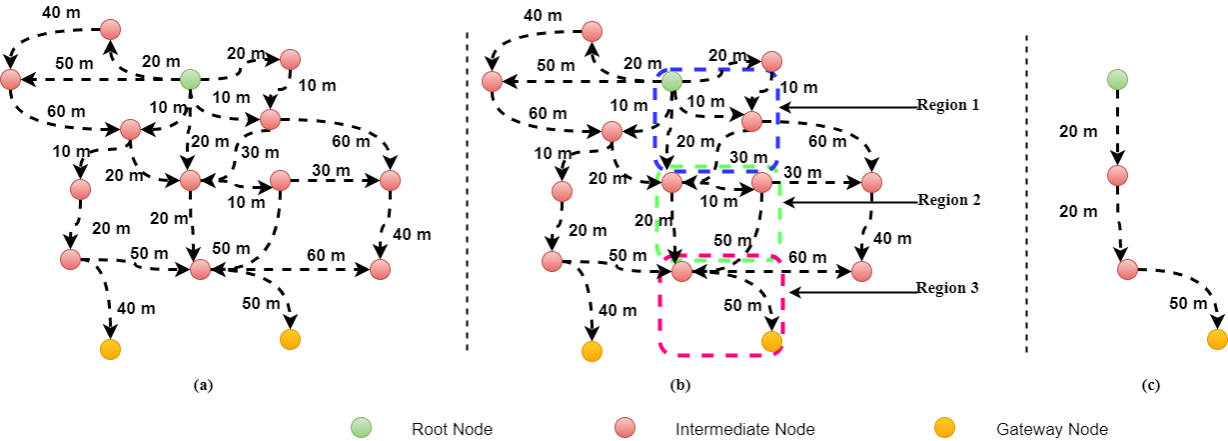
(**a**) SPT implemented without the algorithm; (**b**) STP implemented with the algorithm; and (**c**) optimal path selected.

This type of delay is the time it takes for data packet delivery from its source to its destination (GNs). In a network, nodes are placed in high density areas. This means that there are a lot of links between the MNs and the GNs, which adds distance and steps to communications and causes MNs-to-GNs delays. MNs-to-GNs delay happens when the time it takes for MNs to connect with GNs and the distance between them are both high. In UWSN, more than one path exists between the sender and receiver node. With the help of forwarder selection, paths create in a specific direction, reducing the paths to GNs. Our proposed protocol is elliptical in shape, creating GNs along its trajectory. The nodes make paths towards the trajectory GNs possible. As a result, communication delays between MNs and GNs decrease. Many paths exist between the MNs and GNs, so the SPT has been applied to find the shortest one. MNs calculate the distance toward the GNs, and GNs calculate by using the SPT algorithm as shown in Figure 4. P represents all paths toward the GNs in terms of SP (shortest path).

(1) Num=P

The C-SPT is used to choose the shortest path from all existing paths by using the SPT algorithm.

(2) C-SPT(num)

Suppose that G is a graph, E is both edges, and V is a distance. Set the source node as the current node and mark the rest of the nodes as unvisited nodes. Assess the unvisited nodes for the current node and estimate their distances. Compare the current distances to the calculated distances and assign each V the value corresponding to the shortest distance. When the current node considers all its neighbors, mark them as visited. If the destination source is not available, consider it unvisited. If highlighted, keep it as visited.

## 5. ESEDG Comparison with Existing Routing Protocol

To evaluate the proposed techniques against realistic parameters, we conducted Network Simulator NS-3 version 3.35 (available at: https://www.nsnam.org/releases/ns-allinone-3.35.tar.bz2, accessed on 9 June 2022) for 350 nodes. A 3D environment was established with an area dimension of 1200×1000×1000 with an acoustic link of 1500 m/s and bandwidth of 3 kh. The size of the beacon message was around 54 bits, and other related parameters such as packet size data rates, transmission range, receiving and sending time, and initial and final energy were the same as taken. These parameters are also listed in Table 1. As per acoustic understanding, the node’s movement was only considered in the horizontal direction as vertical movement is not considered the ideal value because the horizontal movement of the node is only 2 m/s in the dedicated acoustic framework that is used for this simulation.

### 5.1. Data Storage and Extraction

ESEDG stores data in a distributed database where a group of sensor nodes act as data sources, storing observed data in rows of a relational manner as discussed in [21,32,33,34]. All rows have been stored in a trace file format and have SQL-like abstractions. AWK script is used to extract data from trace files. It is a method based on data and consists of three sections. The ’BEGIN’ section initializes the variables first. The second section is called ’Content’, and it is where calculations and other procedures are utilized to draw the results from the trace file. The third section, ’END’, is where the results are shown.

### 5.2. Performance Assessment

To evaluate the proposed technique and to measure its performance, we selected the different appropriate benchmark studies Data Gathering Algorithm for Sensor (DSG), Data Gathering Algorithm for the AUV (DGA) [9], AUV-aided Energy-Efficient Data Collection (AEEDCO) and its variant Approximate AEEDCO-A [13], AUV-based location predictors (ALP) [23], and Scalable and Efficient Data Gathering (SEDG) [24]. The AUV-aided Efficient Data Gathering (AEDG) [21] is best suited for comparison. It is interesting to know that both these algorithms are used for data gathering purposes in the underwater environment.

The data gathering classification method with layer swap mechanism is the core value of DSG–DGA. The DSG algorithm is designed for sensors that are the fundamental entity for the collection of data underwater, and DGA is used for the AUV with a purpose the same as DSG. The proposed and the compared approach have focused especially on end-to-end delay reduction.

The other compared approach is the AUV-aided Energy-Efficient Data Collection (AEEDCO) and its variant Approximate AEEDCO-A [13]. It is a data collection protocol that is primarily based on the AUV working scenario. The selection of the AUV path is discussed in detail in the travel salesman problem. Our proposed approach is best described as a path in an elliptical shape and surrounded by a number of gateways with the same objective of reducing AUV traveling time.

Another approach deemed fit for comparison is also the AUV AUV-location predictors (ALP) [23]. This is a data collection scheme to balance the energy consumption in an underwater wireless sensor network. ALP works for the AUV trajectory by sending and receiving data from the AUV to sink and vice versa. The trajectory of the AUV in ALP and the elliptical shape in the proposed approach are the same and provide enough time for receiving and sending data through the AUV directory. Therefore, ALP is considered to prolong the network lifetime and PDR more than the other AUV-based approaches. The justification of these approaches is just AUV-based mechanism and data collection scheme.

The Scalable and Efficient Data Gathering (SEDG) [24] routing protocol with the number of associated Gateways Nodes (GNs) and its selection criteria based on RSSI and Shortest Path Tree (SPT) is restricted to energy efficient. The movement of the AUV underwater is elliptical. Therefore, GNs are dependent according to the quantity of Member Nodes (MNs). The well-known and considered baseline approach in this work includes using the AUV as a central focus for data gathering. The AUV-aided Efficient Data Gathering (AEDG) [21] routing protocol for reliable data delivery is used. In our proposed scenario, we compare AEDG with ESEDG and evaluate path and transmission loss (both are measured in dB), average end-to-end delay, PDR, number of dead nodes, network throughput, and network lifetime, respectively. For evaluation, the experimental setup includes from 50 nodes to 350 with a 3D environmental area of 1200×1000×1000 m${}^{3}$. The initial energy is 100 J, which can increase or decrease during simulation with increments in the transmission range. For this experiment, the transmission range was 200 m.

### 5.3. Performing Assessment

In this phase, we compared the performance of ESEDG with DSG–DGA (sensor and AUVs oriented), ALP (location predictor and trajectory time of the AUV), SEDG (gateways oriented), and AEDG (PDR, E2E delay, throughput oriented), respectively. The previous section already described the justification of all these protocols above. The realistic simulation parameters are average E2E delay and PDR analysis, network throughput, network lifetime, number of dead nodes, and energy consumption.

#### 5.3.1. Analysis of Average E2E Delay and PDR

The AUV is the medium from source to destination for any AUV-oriented routing protocols. The delay cycle is normal and natural when the data collection process is initiated through AUVs. The delay occurs when data moves from the source node and GNs to the AUV for further processing and destination nodes. The time of the AUV trajectory also plays a role in unnecessary delays. For 50 nodes, the proposed protocol ESEDG has an elliptical circuit of the AUV from source to destination; therefore, it remains 0.3 s until the nodes reach 150. Similarly, the E2E delay decreased from 0.3 s to 0.2 s for 200 nodes compared to ALP.

**Figure 5:**
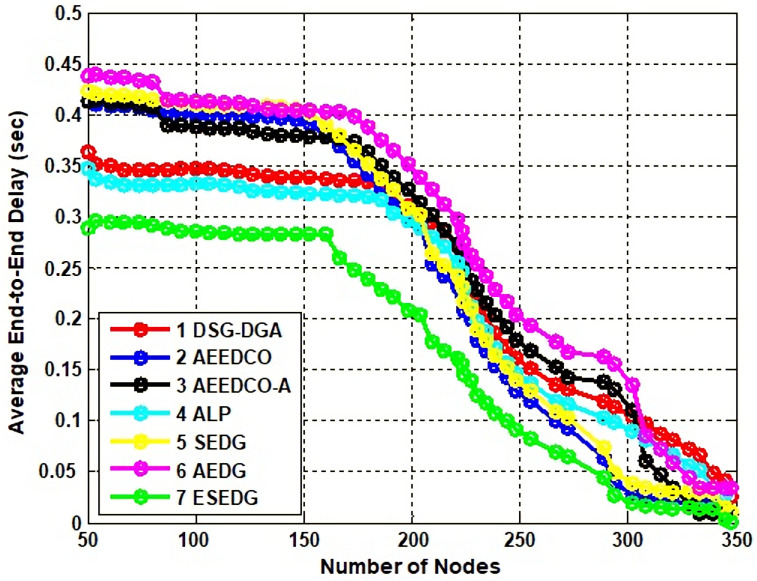
Analysis of E2E delay with baseline schemes.

DSG–DSA is closer to the proposed approach with the working mechanism of the AUV cycle but has a 0.35 s and a 0.38 s delay, which shows it is not outperformed in the comparison stage. Meanwhile, E2E delay is the fundamental concern of AEDG. Due to the non-elliptical path in AEDG (only typical trajectory of the AUV is being examined) and because of the large rounds of the AUVs that unnecessarily delay the traffic burden of the intermediate nodes, the delay goes to 0.44 s. Furthermore, AEEDCO and AEEDCO-A are both used for the AUVs reduction in travel time, but despite the path planning, it resulted in an exceedingly average E2E delay. The primary comparison of the proposed approach ESEDG with other benchmarks is 11% increased for AEDG, 9% for SEDG, and 6% for ALP. In contrast, other techniques such asAEEDCO and AEEDCO-A have almost the same number of E2E delays which are 0.41 and 0.40 s, respectively, as represented in Figure 5.

In contrast, the Packet Delivery Ratio (PDR) has an inversely proportional relationship with E2E; as PDR increased, E2E delay decreased and vice versa (this condition only hold when other networks’ condition remains stable). A PDR is only used when a large number of nodes is running and impacting the network. Initially, no impact is recorded until the nodes reach 35. When all the nodes are in a working state, the impact of the PDR for the proposed approach and EDG is about 46% and 48%, respectively. Meanwhile, the rest of the baseline schemes such as SEDG have 4% lower than ESEDG, and AEEDCO-A has almost the same values as ESEDG when it reaches 46%.

#### 5.3.2. Impact of Path and Transmission Loss Analysis

**Figure 6:**
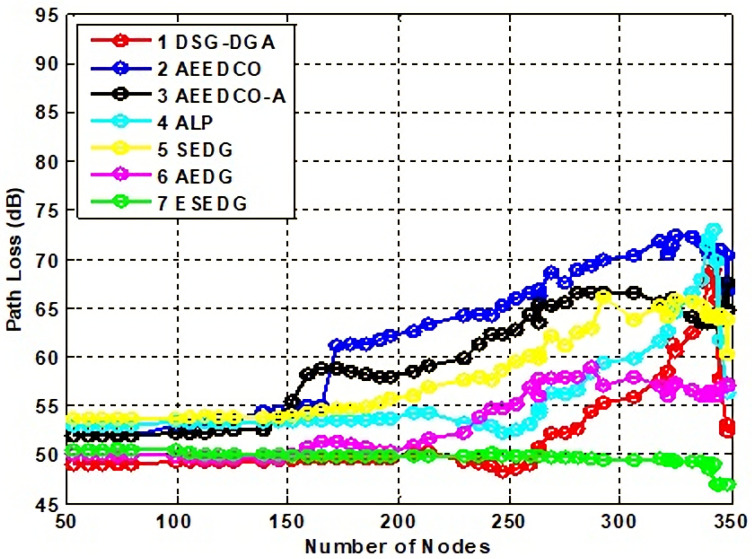
Analysis of path loss with baseline schemes.

During the the AUV journey, a common underwater scenario might be that the transmission and path are disturbed. During the data collection process, the AUV is monitoring the GNs and MNs as data is loaded. The AUV is surrounded by MNs, so the path between the AUV–AUV communication may be disturbed. To evaluate such a situation, we compare our proposed technique with the base scheme AEDG (AUV path are used), SEDG (saleable path are used), ALP (path location indicator is present), AEEDCO, and AEEDCO-A. Path planning and trajectory are analyzed, and the DSG–DGA (sensor and AUV data gathering mechanism are used) are compared. The path loss is measured in dB, and it is only 50 dB for 50 to 60 nodes, while for 300 plus nodes, it decreases further by 10 dB and reaches only 4 dB for ESEDG. Meanwhile, for other techniques, such asAEDG, it reaches 58 dB, and for SEDG it further increases to 65 dB. SEDG has a very long trajectory to complete the path; therefore, it can gradually increase. As for the concern about the location indicator, ALP goes to a maximum value of 72 dB which is slightly increased for AEEDCO-A to 2 dB, and DSG–DGA has 11 dB.. Path loss comparison between ESEDG and other techniques is shown in Figure 6.

**Figure 7:**
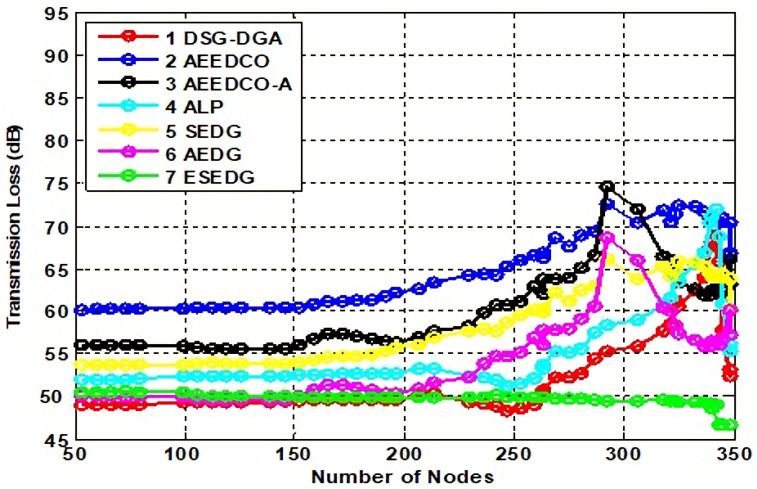
Analysis of transmission loss with baseline schemes.

In Figure 7, the transmission loss analysis has the same value with respect to path loss analysis as described earlier. Transmission loss was as follows: for DSG–DGA 68 dB, for AEEDCO 71 dB, for AEEDCO-A 74 dB, and for ALP 70 dB. These values lie between DSG–DGA and AEEDCO, respectively. Similarly, SEDG and AEDG have 60 dB to 65 dB for transmission loss analysis, while the proposed technique outperformed all with only 46 dB.

#### 5.3.3. Analysis of Network Throughput and Lifetime with Number of Dead Nodes and Energy Consumption

**Figure 8:**
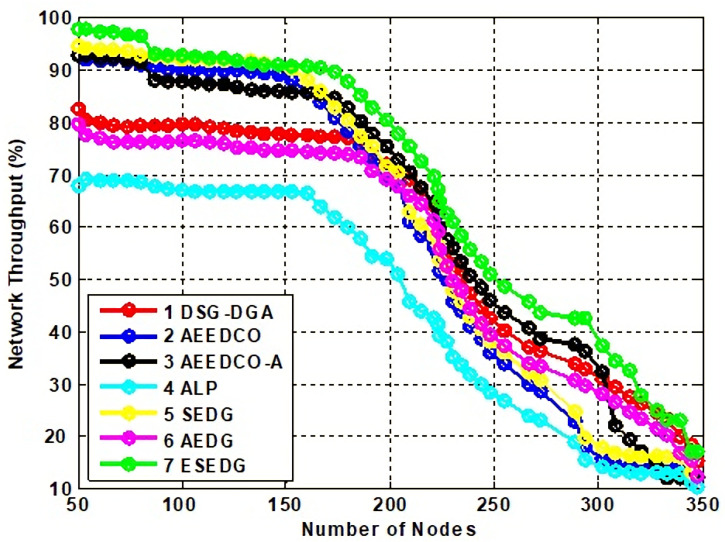
Analysis of network throughput loss with baseline schemes.

**Figure 9:**
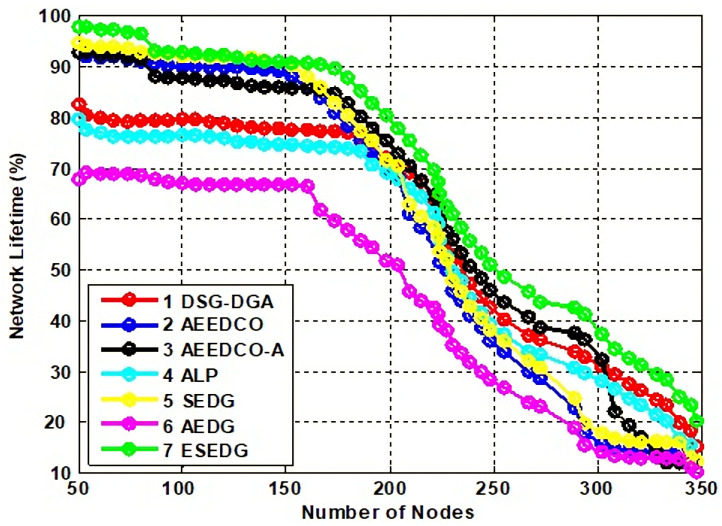
Analysis of network lifetime loss with baseline schemes.

**Figure 10:**
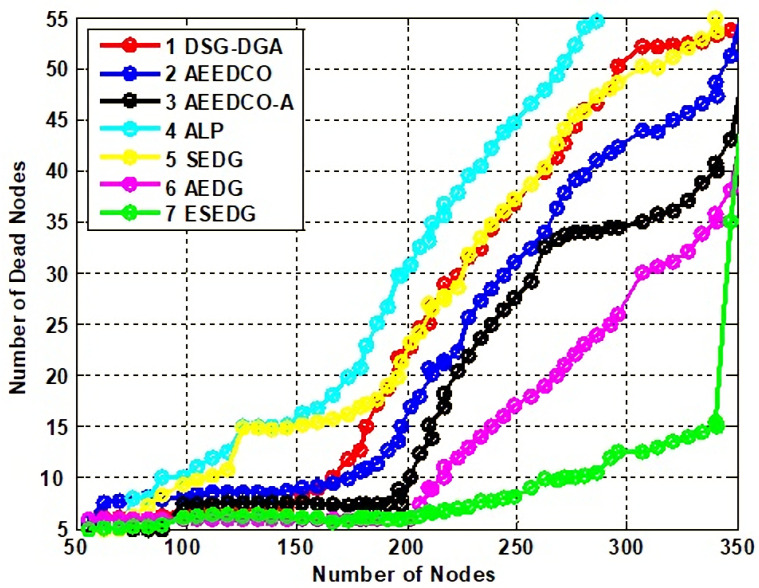
Analysis of network dead nodes with baseline schemes.

**Figure 11:**
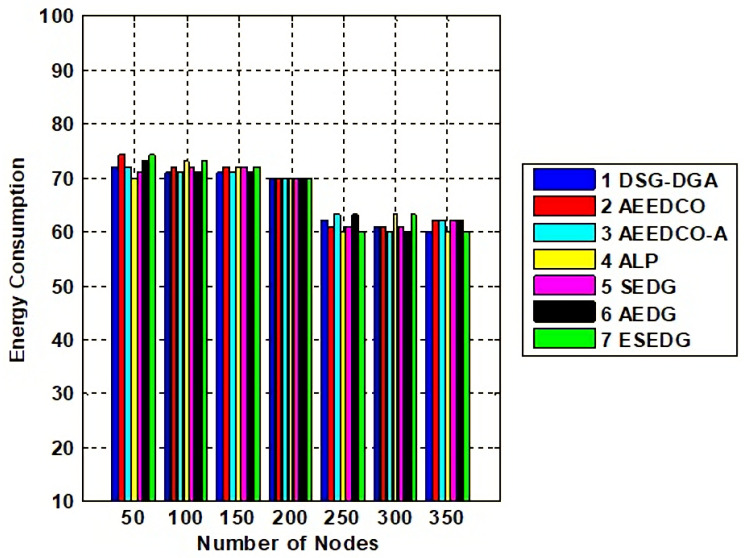
Analysis of network energy consumption with baseline schemes.

Network lifetime and its throughput are the fundamental characteristics used to evaluate the performance of the schemes under study. Network throughput for compared techniques is shown in Figure 8. Network lifetime due to an elliptical path would be recorded as maximum which is 80 % compared to other schemes that have values below the proposed scheme. ESEDG outperformed other baseline schemes as shown in Figure 9. The simulation recorded network throughput analysis as both are directly proportional to each other. In addition, we also evaluated the secondary parameters that ensure the node’s quantity before and after network deployment. Reanalysis performed with a number of dead nodes validates the working of ESEDG with its pros and cons. Figure 10 represents the detailed analysis of the number of dead nodes against all baseline schemes, which shows that the ESEDG has the lowest dead nodes ratio. In Figure 11, the overall energy consumption of the network under different routing protocols is shown. Initially, the energy consumption of ESEDG is higher compared to other protocols due to the routing path association of MN and GN. Once paths are created, their energy consumption level decreases compared to other techniques.

## 6. Conclusions

In this paper, we have proposed ESEDG for maximum data gathering to resolve the acoustic environment’s challenges such as slow throughput, transmission loss (dB), average end-to-end delay, and high energy consumption for UWSNs. The ESEDG protocol gathers data by using a center-positioned node for the suboptimal horizontal trajectory for the movement of the AUV. The ESEDG improves the network life, throughput time, imbalanced energy consumption, and end-to-end delay with the help of a maximum number of GNs. Network lifetime and throughput due to elliptical path would record the maximum which is 80% compared to other schemes. The ESEDG outperformed baseline schemes as shown in the output of the simulation in which the proposed system achieved the targets of balanced energy consumption and network delay. The ESEDG protocol was evaluated using NS3 version 3.35 with 350 nodes. The simulation was performed to evaluate the performance of the proposed protocol compared to the existing protocol AEDG, and our protocol achieved the target performance.

In future, we will make ESEDG more energy-efficient and support multiple AUVs for the data gathering process. Furthermore, we will also take into account state-of-art approaches such as mobile edge computing and software-defined network techniques for reducing the computation costs of the networks.

## Figures and Tables

**Table 1:** Simulation settings and their values.

Simulation Parameters	Values
Number of nodes	350
Network range	1200×1000×1000
Number of AUV	1
Number of sink nodes	1
Energy	Energy Model
Initial energy	100 J
Acoustic network speed	1500 m/s
Communication medium	Wireless
Wireless channel	Radio and Acoustic
Frequency	15 KHz
Transmission range	200 m
Receiving	0.6 W
Data packet size	50 B
Beacon message size	54 B
Data rates	12 kbps
Mobility	static and random
Transmission Power	0.5 W
Idle Power	0.008 W
Sleeping Power	0.01
Physical Layer	UnderwaterPhy
Mac Layer	UnderwaterMac
Antenna	OmniAntenna

## Data Availability

Not applicable.
